# Effects of ingesting JavaFit Energy Extreme functional coffee on aerobic and anaerobic fitness markers in recreationally-active coffee consumers

**DOI:** 10.1186/1550-2783-4-25

**Published:** 2007-12-08

**Authors:** Michael D Roberts, Lemuel W Taylor, Jennifer A Wismann, Colin D Wilborn, Richard B Kreider, Darryn S Willoughby

**Affiliations:** 1 Department of Health and Exercise Science, University of Oklahoma, Norman, OK 73069, USA; 2 Department of Health, Leisure, and Exercise Science, University of West Florida Pensacola, FL 32514, USA; 3 Department of Health, Human Performance, and Recreation, Baylor University Waco, TX 76798, USA; 4 Department of Exercise and Sport Science, University of Mary Hardin-Baylor Belton, TX 76513, USA

## Abstract

The purpose of this study was to examine the effects of ingesting JavaFit™ Energy Extreme (JEE) on aerobic and anaerobic performance measures in recreationally-active male and female coffee drinkers. Five male (27.6 ± 4.2 yrs, 93.2 ± 11.7 kg, 181.6 ± 6.9 cm) and five female (29 ± 4.6 yrs, 61.5 ± 9.2 kg, 167.6 ± 6.9 cm) regular coffee drinkers (i.e., 223.9 ± 62.7 mg·d^-1 ^of caffeine) participated in this study. In a cross-over, randomized design, participants performed a baseline (BASELINE) graded treadmill test (GXT) for peak VO_2 _assessment and a Wingate test for peak power. Approximately 3–4 d following BASELINE testing, participants returned to the lab for the first trial and ingested 354 ml of either JEE or decaffeinated coffee (DECAF), after which they performed a GXT and Wingate test. Criterion measures during the GXT included an assessment of peakVO_2 _at maximal exercise, as well as VO_2 _at 3 minutes and 10 minutes post-exercise. Additionally, time-to-exhaustion (TTE), maximal RPE, mean heart rate (HR), mean systolic pressure (SBP), and mean diastolic blood pressure (DBP) were measured during each condition. Criterion measures for the Wingate included mean HR, SBP, DBP, peak power, and time to peak power (TTP). Participants then returned to the lab approximately one week later to perform the second trial under the same conditions as the first, except consuming the remaining coffee. Data were analyzed using a one way ANOVA (p < 0.05). Our results indicate that JEE significantly increased VO_2 _at 3 minutes post-exercise when compared to BASELINE (p = 0.04) and DECAF (p = 0.02) values, which may be beneficial in enhancing post-exercise fat metabolism.

## Introduction

Endurance athletes commonly ingest caffeine-laden supplements with the intent of improving sports performance. JavaFit™ Energy Extreme (JEE, Javalution Coffee Co, Fort Lauderdale, FL) is a functional gourmet coffee that contains a proprietary blend of caffeine, garcinia cambogia, chromium polynicotinate, and citrus aurantium, and is marketed to increase energy expenditure. Our lab has previously shown JEE to acutely increase resting energy expenditure levels in males and females [1]. Furthermore, Hoffman and colleagues [2] have demonstrated that while JEE does not affect anaerobic peak power, it did significantly increase time to exhaustion during submaximal cycling. However, it is currently unknown if supplementing with JEE acutely enhances maximal aerobic exercise performance. Furthermore, little is still known concerning the effects that JEE has on anaerobic peak power.

Other research has evaluated the efficacy of some of the aforementioned ingredients present in JEE in regards how each of them improves aerobic and/or anaerobic performance. Caffeine ingestion has been reported to improve time to exhaustion and work output during endurance exercise [3-7]. Additionally, the ergogenic effects of caffeine in regards to endurance performance have been observed with dosages as low as 3 mg·kg^-1 ^body mass [4]. However, reports suggesting that caffeine enhances anaerobic performance have been equivocal. Doherty *et al*. [8] demonstrated that ingesting caffeine prior to exercise elicits a greater improvement in endurance exercise when compared to short-term exercise. Conversely, a related study [9] reported that high-intensity cycling performance can be increased following moderate caffeine ingestion (i.e., 5 mg·kg^-1^). Thus, while caffeine may improve anaerobic performance as a result of central nervous system (CNS) stimulation and/or catecholamine secretion [10], equivocal findings in regards to the ergogenic benefits of caffeine during anaerobic exercise confound this hypothesis.

Garcinia cambogia is an herbal ingredient that contains (-)-hydroxycitric acid ((-)-HCA) and is present in many thermogenic supplements due to its ability to reduce *de novo *fatty acyl synthesis. Shara *et al*. [11] demonstrated that supplementing rats with (-)-HCA over a 90 day period caused a significant reduction in body weight. Regardless, little is known concerning the ergogenic effects of Garcinia cambogia. Similarly, chromium polynicotinate has been clinically investigated for its anti-hypertensive properties as well as its ability to reduce lipid peroxidation after long-term supplementation in rats [12]. However, little research has studied the effects that chromium polynicotinate has on exercise performance. Citrus aurantium is also a commonly found ingredient in thermogenic aids due to its action as a β_3 _receptor-agonist to potentially increase lipolysis and thermogenesis [13]. Specifically, citrus aurantium contains the beta-agonists oxedrine (synephrine) and octopamine, which are structurally similar to epinephrine, and is postulated to act by way of a β_3 _receptor mediated mechanism with no cardiovascular effects [14].

It has previously revealed that synephrine is approximately 3.5 times as effective in stimulating lipolysis as octopamine [15]. More recently, studies have shown that both octopamine and synephrine appear particularly effective in stimulating lipolysis [16]. Nonetheless, limited evidence exists examining the ergogenic effects that citrus aurantium has during aerobic and/or anaerobic exercise. As mentioned previously, Hoffman and colleagues [2] has addressed the ergogenic effect that JEE has on aerobic and/or anaerobic parameters in humans. The main finding from the abovementioned study was that JEE significantly improved time to exhaustion during a prolonged cycling bout (JEE: 35.3 ± 15.2 min, PLA: 27.3 ± 10.7 min, p < 0.05). Thus, while the exercise effects of JEE have been studied during submaximal exercise bouts, it is currently unknown how JEE affects maximal aerobic and/or anaerobic performance. Therefore, the purpose of this study was to examine the effects that JEE has on maximal aerobic performance in healthy, college-aged individuals that regularly consume caffeine. Furthermore, we sought to also examine how JEE affects anaerobic exercise performance measures during a Wingate cycle ergometer test.

## Methods

### Study design

**Figure 1:**
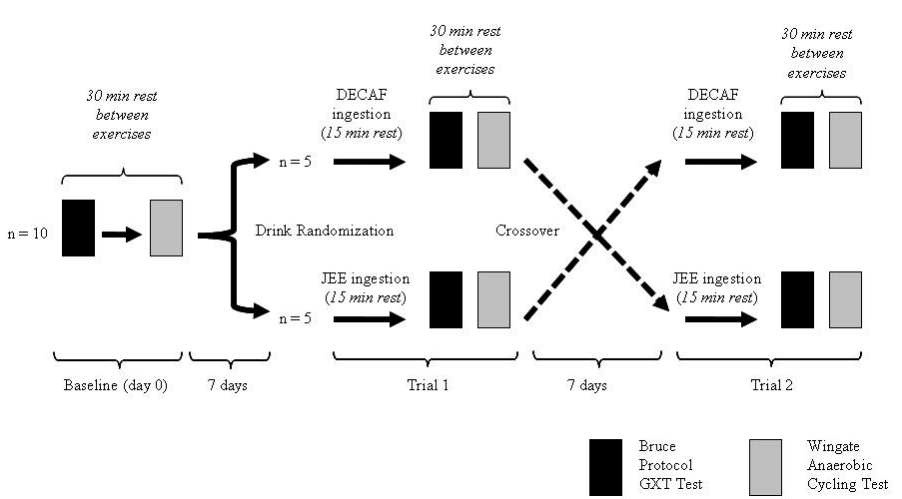
The crossover design employed during this study.

This study employed a single-blind, randomized, crossover design, whereby participants performed two exercise trials (i.e., trial 1 vs. trial 2) separated by one week to assess aerobic and anaerobic parameters after consuming either JEE or DECAF (Figure 1). It should be noted that prior to trial 1, participants performed the standardized exercise protocol without consuming the supplement (BASELINE). Fifteen minutes following supplement ingestion during both trials participants performed a graded treadmill test (GXT) and Wingate power test. Criterion measures assessed for the GXT included peak VO_2 _maximal exercise, mean HR, SBP, and DBP, time to exhaustion (TTE), and VO_2 _at 3 minutes and 10 exercise (representing excess post-exercise oxygen consumption (EPOC)). Criterion measures for the Wingate test included mean HR, SBP, and DBP, mean and peak power, and time to peak power (TTP).

### Participants

Healthy, recreationally-active males (n = 5, 27.6 ± 4.2 yrs, 93.2 ± 11.7 kg, 181.6 ± 6.9 cm) and females (n = 5, 29.0 ± 4.6 yrs, 61.5 ± 9.2 kg, 167.6 ± 6.9 cm) that were self-reported coffee drinkers (i.e., 223.9 ± 62.7 mg·d^-1 ^of caffeine from self-reported logs) served as participants in this study. Participants underwent a mandatory medical screening by a research nurse to determine whether he/she met entry criteria to participate in the study. Individuals considered low-to-moderate risk for cardiovascular disease with no contraindications to exercise as outlined by the American College of Sports Medicine (ACSM) [17] were allowed to participate in the study. Furthermore, individuals could not have consumed any nutritional supplements (excluding multi-vitamins) one week prior to the study and it was required that participants consumed at least 100 mg·d^-1 ^caffeine (i.e., equivalent to one cup of coffee) daily in order to be eligible for participation. Participants meeting the eligibility criteria were informed of the requirements of the study and signed an informed consent statement in compliance with the Human Subjects Guidelines of Baylor University and ACSM guidelines [17].

### Baseline aerobic and anaerobic exercise testing (day 0)

Participants reported to the lab for BASELINE between the hours of 0800–1000 for baseline testing following a 10-hour fast. Participants remained seated for 15 minutes prior to baseline HR and SBP/DBP determination. Participants then performed a GXT employing the Bruce protocol [17]. Each participant was told to stop the test once volitional fatigue was attained. VO_2 _was determined using indirect calorimetry (True One 2400 Metabolic Measurement System, ParvoMedics Inc., Sandy, UT) during all 3-minute stages of the GXT as well as 3 and 10 minutes following the cessation of exercise for EPOC determination. Parameters including HR, SBP, and DBP were assessed using standard procedures during each stage as well as 3 and 10 minutes following exercise and averaged to attain mean values. Maximal RPE was assessed by having each participant place his/her finger on a Borg RPE scale immediately following the cessation of exercise.

Following a 20-minute rest period, participants performed a 30-second Wingate protocol (Lode Excalibur, Lode, Groningen, The Netherlands). This test consisted of having each participant sprint in an all out fashion on the bicycle ergometer for 30 seconds against a standard workload of 0.075 kgkg^-1 ^of body weight. Parameters including HR, SBP, and DBP were assessed using standard procedures prior to as well as 3 and 10 minutes following exercise and averaged to attain mean values. Integrated software (Wingate version 1.0.7, Lode, Groningen, The Netherlands) determined other criterion variables including mean and peak power, and TTP. Test-retest reliability assessment for studies conducted in our laboratory have yielded high values for mean power (*r *= 0.95) and peak power (*r *= 0.69).

### JEE versus DECAF trials

Participants returned to the lab one week following day 0 to complete trial 1. Participants remained seated for 15 minutes prior to baseline HR, SBP, and DBP determination. Participants were then randomly assigned to ingest 354 ml of drip-brewed coffee containing either JEE (450 mg of caffeine, 1200 mg of garcinia cambogia, 360 mg of citrus aurantium extract, and 225 mcg of chromium polynicotinate) or DECAF within a 1-minute time period. Fifteen minutes following drink consumption participants performed the GXT followed by the Wingate anaerobic peak power test in the same manner as during BASELINE. In a cross-over fashion, participants returned to the lab 1 wk following trial 1 to perform trial 2. During trial 2, each participant abided by the same drink ingestion and standardized exercise procedures with the exception being that he/she consumed the drink that was not consumed during trial 1.

### Dietary analysis

Subjects were required to keep dietary records 24 hours prior to each trial. Dietary analysis was performed using the Food Processor III Nutrition Software package (ESHA Research Inc., Salem, OR) to ensure between-subject caloric homogeneity between trials.

### Statistical analysis

All statistical procedures were performed using the SPSS 14.0 statistical package (SPSS Inc, Chicago, IL). All criterion variables during the Wingate and Bruce GXT tests were analyzed between the baseline, DECAF, and JEE conditions using separate one-way ANOVAs. Caloric intake between treatments was also compared using a one-way ANOVA. When a significant between-treatment effect was present a Tukey's *post hoc *analysis was used to determine where pair-wise differences existed.

## Results

### Side effects and caloric intake between sessions

No adverse side effects were reported throughout the study as both the supplementation and exercise regimens seem to be well tolerated. In addition, no between-session differences were found to exist concerning caloric intake (p > 0.05).

### Aerobic parameters during the bruce GXT

**Figure 2:**
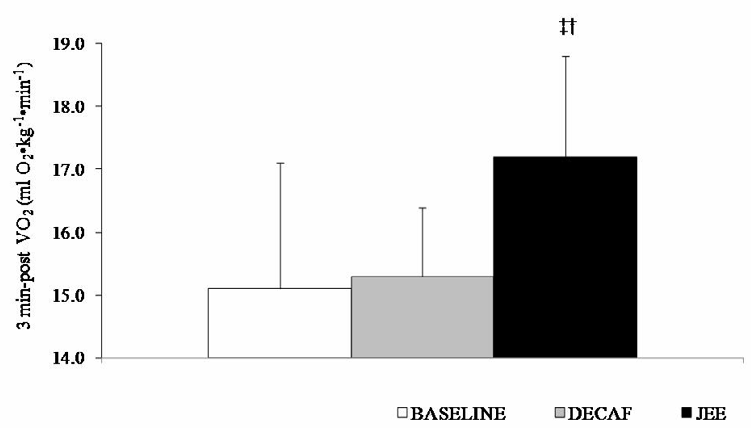
Between-treatment 3-minute post VO_2 _values. Values expressed as means ± SD. ‡JEE > BASELINE, p = 0.02. †JEE > DECAF, p = 0.04. BASELINE: day 0 (no supplement), DECAF: decaffeinated coffee, JEE: JavaFit™ Energy Extreme.

JEE significantly increased 3 minutes post VO_2 _(i.e., EPOC) when compared to BASELINE (p = 0.02) and DECAF (p = 0.04) (Figure 2). JEE also significantly elevated mean HR compared to DECAF (p = 0.03) during the GXT. *Post hoc *analysis revealed that heart rate elevations tended to be at the point of volitional fatigue (JEE: 187 ± 5 vs. DECAF 180 ± 7 beatsminute^-1^, p = 0.08) and during the 3-minute recovery period (JEE: 133 ± 9 vs. DECAF 117 ± 15 beatsminute^-1^, p = 0.03). However, there were no significant differences between the mean HR values during the JEE and BASELINE treatments (p = 0.65). No significant differences between treatments were observed in regards to other criterion measures (Table 1).

**Table 1:** Comparison of hemodynamic and performance variables during wingate test

Parameter	Baseline	DECAF	JEE	Significance
Mean HR (beatsmin^-1^)	111 ± 38	110 ± 39	114 ± 42	0.44
Mean SBP (mmHg)	127 ± 18	131 ± 21	128 ± 19	0.69
Mean DBP (mmHg)	67 ± 8	66 ± 10	68 ± 10	0.78
Mean Power (W)	657 ± 229	655 ± 265	649 ± 242	0.99
Max Power (W)	1291 ± 431	1343 ± 508	1321 ± 479	0.97
TPP (s)	3.9 ± 0.1	3.8 ± 0.3	3.8 ± 0.2	0.52

Values are expressed as means ± SD.

TTP: time to maximal power.

### Anaerobic parameters during the wingate test

All anaerobic performance variables are presented in Table 2. No significant differences between treatments were observed in regards to all of the criterion measures during the Wingate test (p > 0.05).

**Table 2:** Comparison of hemodynamic and performance variables during bruce GXT

Parameter	Baseline	DECAF	JEE	Significance
Mean HR (bpm)^‡^	112 ± 8	107 ± 7	115 ± 6	0.03
Mean SBP (mmHg)	124 ± 7	123 ± 8	129 ± 12	0.36
Mean DBP (mmHg)	69 ± 9	70 ± 8	70 ± 9	0.60
TTE (min)	14.2 ± 1.1	14.4 ± 1.1	15.0 ± 1.0	0.17
Max RPE	17.7 ± 1.2	17.9 ± 1.1	18.6 ± 1.2	0.14
Peak VO_2 _(ml·kg^-1^·min^-1^)	46.8 ± 6.5	46.4 ± 4.1	48.6 ± 4.4	0.59
3 min post VO_2 _^‡† ^(ml·kg^-1^·min^-1^)	15.1 ± 2.0	15.3 ± 1.0	17.2 ± 1.6	0.02
10 min post VO_2 _(ml·kg^-1^·min^-1^)	5.3 ± 1.3	5.8 ± 1.2	6.3 ± 0.6	0.16
Max RER (VCO_2_·VO_2_^-1^)	1.13 ± 0.06	1.16 ± 0.08	1.16 ± 0.06	0.56

Values are expressed as means ± SD.

Mean HR: mean heart rate, Mean SBP: mean systolic blood pressure, Mean DBP: mean diastolic blood pressure, TTE: time-to-exhaustion, Max RPE: maximal rate of perceived exertion, peak VO_2_: maximal oxygen uptake

‡JEE > DECAF, p < 0.05

†JEE > BASELINE, p < 0.05

## Discussion

This is the first study to investigate the effects that JEE exerted on maximal aerobic parameters during a Bruce GXT. Furthermore, this study confirms the findings from Hoffman et al. [2] concerning the effects that JEE exert on anaerobic exercise performance parameters during a Wingate test. The main finding from this study was that JEE increases 3 minutes post-exercise VO_2 _levels in young, college-aged individuals. However, it appears that JEE does not acutely enhance aerobic and/or anaerobic parameters during maximal exercise.

### Effects of JEE on aerobic performance

While past research contends that caffeine-laden supplements are efficacious in increasing TTE during submaximal aerobic exercise bouts [18], JEE did not appear to acutely enhance parameters of aerobic fitness during a maximal Bruce GXT. JEE did, however, significantly elevate post-exercise oxygen consumption (EPOC). EPOC is the amount of oxygen needed to resynthesize intramuscular high energy phosphates and glycogen following a prolonged bout of high intensity exercise [19]. Furthermore, EPOC has been postulated to be a thermogenic process whereby fatty acids are oxidized in order to replenish the abovementioned fuel stores [19]. EPOC can be elevated due to exercise-induced increases in thermogenic hormones including catecholamines following exercise [20]. Although the humoral response to each treatment was not assessed, JEE may have synergistically increased circulating catecholamines which could have contributed to the increases seen in the 3 minute post-exercise VO_2 _values. Moreover, we are in agreement with past literature demonstrating that moderate (i.e., 5 mg·kg^-1^) and high doses (i.e., 10 mg·kg^-1^) of caffeine increases EPOC following prolonged cycling at 55% VO_2max _[20]. Authors from the aforementioned investigation attributed greater levels in EPOC to be caused by the lipolytic effects of caffeine. Furthermore, other evidence suggests that an elevation in free fatty acids following exercise is associated with greater EPOC levels [21]. While the current finding differs from the findings of Hoffman and colleagues [2] who reported no differences in EPOC up to 30 minutes following exercise, the same research group did find that JEE exhibited a thermogenic effect during a 150 min resting period in seven responders of the supplement [22]. Thus, it may be possible that consuming JEE prior to a more prolonged and less intense steady-state aerobic exercise bout does not further enhance EPOC due to a lessened catabolism of high energy phosphate stores when compared to a maximal intensity exercise bout.

Participants undergoing the JEE trial experienced trend increases in TTE during the Bruce GXT (p = 0.17). As mentioned previously, past literature [10] suggests that caffeine ingestion enhances glycolysis in exercising muscle through increases in circulating epinephrine. It has been well established that glycolysis-dependent ATP production enables exercising individuals to sustain a greater power output over short time periods [19]. Thus, potential JEE-induced increases in glycolysis may have contributed to the trend TTE increases during the Bruce GXT. However, this hypothesis remains untested due to the fact that we did not quantify circulating epinephrine, lactate, and/or intramuscular glycogen levels during or after the Bruce GXT. The observed TTE trend in this study is paralleled by the findings of Hoffman and colleagues [2] who did observe a significant increase in time to exhaustion during prolonged cycling bouts at 75% VO_2max_. However, it should be noted that both the exercise modality and intensity differed between studies.

Participants undergoing the JEE trial also experienced trend increases in maximal RPE during the Bruce GXT (p = 0.14). A recent meta-analysis by Doherty and Smith [8] analyzed 21 studies that quantified RPE with or without caffeine during different exercising conditions. While the all of the caffeine treatments exhibited a significantly reduced mean RPE compared to placebo treatment, maximal RPE values during exercises to exhaustion did not differ from placebo values. Thus, while JEE may reduce RPE during submaximal exercise, our finding concerning the lack of change in maximal RPE levels is in agreement with the data presented by the aforementioned authors.

### Effects of JEE on anaerobic performance

Past research contends that ingesting caffeine-laden supplements may increase CNS stimulation by blocking inhibitory adenosine receptors and/or altering the intracellular ionic environment, both effects which increase neuronal excitability [23]. However, contrary to previous reports suggesting that caffeine enhances anaerobic capacity [24], JEE does not appear to acutely enhance anaerobic performance parameters during a maximal exercise bout. We are in agreement with past studies demonstrating that ingesting 5–7 mg·kg^-1 ^of caffeine up to 60 minutes prior to exercise did not enhance anaerobic capacity and/or peak power during a Wingate test [10,25]. We may have not observed between-treatment differences due to the fact that the caffeine dosage was not high enough to elicit positive performance increases. JEE contains 450 mg caffeine which provided a significantly greater dose to females when controlling for body mass (males: 5.2 mg·kg^-1 ^vs. females: 8.0 mg·kg^-1^, p < 0.001). It should be noted, however, that further statistical analysis revealed no significant differences in any of the criterion variables between treatments when controlling for gender. Therefore, it appears that the caffeine dosage provided by JEE was not adequate in enhancing maximal anaerobic performance regardless of body mass. It also appears that the other active ingredients in JEE may not be efficacious in synergistically enhancing anaerobic capacity. Moreover, we are in agreement with the findings of Hoffman and colleagues [2] which stated that JEE did not increase any of the power performance measures when compared to a placebo, decaffeinated beverage.

## Conclusion

Although JEE does not appear to affect maximal aerobic or anaerobic parameters, results from this study suggest that JEE increases short-term EPOC following maximal aerobic exercise. Furthermore, while JEE does elevate heart rate due to its sympathomimetic ingredients, it does not appear to adversely affect hemodynamic markers (i.e., cause an irregularity in heart beat or elevate blood pressure) and/or elicit negative side effects at rest or during maximal exercise bouts. Whether higher doses of JEE affects maximal exercise parameters have yet to be determined.

## Competing interests

The author(s) declare that they have no competing interests.

## Authors' contributions

MDR assisted in data acquisition, statistical analysis, and writing of the manuscript. LWT, JAW, and CDW assisted in the data acquisition. RBK assisted with oversight, lab personnel, and in the statistical analysis. DSW conceived the study, developed the study design, secured the funding for the project, assisted and provided oversight for all data acquisition and statistical analysis, assisted in drafting the manuscript, and served as the faculty mentor for the project. All authors have read and approved the final manuscript.
